# Insights into arsenic multi-operons expression and resistance mechanisms in *Rhodopseudomonas palustris* CGA009

**DOI:** 10.3389/fmicb.2015.00986

**Published:** 2015-09-17

**Authors:** Chungui Zhao, Yi Zhang, Zhuhua Chan, Shicheng Chen, Suping Yang

**Affiliations:** ^1^Department of Bioengineering and Biotechnology, Huaqiao UniversityXiamen, China; ^2^State Key Laboratory Breeding Base of Marine Genetic Resource, Third Institute of Oceanography, State Oceanic AdministrationXiamen, China; ^3^Department of Microbiology and Molecular Genetics, Michigan State UniversityEast Lansing, MI, USA

**Keywords:** *Rhodopseudomonas palustris*, operons, arsenic resistance, regulation

## Abstract

Arsenic (As) is widespread in the environment and causes numerous health problems. *Rhodopseudomonas palustris* has been regarded as a good model organism for studying arsenic detoxification since it was first demonstrated to methylate environmental arsenic by conversion to soluble or gaseous methylated species. However, the detailed arsenic resistance mechanisms remain unknown though there are at least three arsenic-resistance operons (*ars1, ars2*, and *ars3*) in *R. palustris*. In this study, we investigated how arsenic multi-operons contributed to arsenic detoxification in *R. palustris*. The expression of *ars2* or ars3 operons increased with increasing environmental arsenite (As(III)) concentrations (up to 1.0 mM) while transcript of *ars1* operon was not detected in the middle log-phase (55 h). *ars2* operon was actively expressed even at the low concentration of As(III) (0.01 μM), whereas the *ars3* operon was expressed at 1.0 μM of As(III), indicating that there was a differential regulation mechanism for the three arsenic operons. Furthermore, *ars2* and *ars3* operons were maximally transcribed in the early log-phase where *ars2* operon was 5.4-fold higher than that of *ars3* operon. A low level of *ars1* transcript was only detected at 43 h (early log-phase). Arsenic speciation analysis demonstrated that *R. palustris* could reduce As(V) to As(III). Collectively, strain CGA009 detoxified arsenic by using arsenic reduction and methylating arsenic mechanism, while the latter might occur with the presence of higher concentrations of arsenic.

## Introduction

Arsenic (As) is a highly toxic, carcinogenic, clastogenic and teratogenic metalloid (Slyemi and Bonnefoy, 2012). Arsenic occurs primarily as inorganic forms of pentavalent arsenate As(V) and trivalent arsenite As(III), with the latter being regarded as the most mobile and toxic form (Yang et al., 2012). Arsenicals generated from natural and anthropogenic sources are the widely distributed contaminants of freshwater, groundwater and seawater (Stolz et al., 2010; Slyemi and Bonnefoy, 2012; Rodríguez-Lado et al., 2013). Combustion of fossil fuels, mining, and applications of arsenic-containing pesticides/herbicides account for most of the arsenic pollution sources (Stolz and Oremland, 1999). Microorganisms are the principal drivers of arsenic chemical speciation by redox transformations and influence arsenic mobility and toxicity. Most of bacteria and archaea virtually carry arsenic-resistance (*ars*) genes that potentially confer resistance to As(V) and/or As(III) (Jackson and Dugas, 2003). The phenomenon (widespread occurrence of *ars* genes) indicates the ubiquitous distribution of this toxic metalloids in nature.

Some of microorganisms have a significant impact on the biogeochemical transformations of arsenic and are considered as bioremediation reagents for arsenic contamination (Zhang et al., 2015). Increasing interests have been drawn to investigate microbial detoxification of arsenic compounds, particularly to study the arsenic metabolic mechanisms. Furthermore, some microorganisms such as chemoautotrophic arsenite oxidizers oxidize As(III) to As(V) to gain energy for cell growth when fixing inorganic carbon (CO_2_). Instead, microbes that use As(V) as an electron acceptor in anaerobic respiration lead to the production of As(III), indicating that arsenic plays various roles in microbial metabolism (Heinrich-Salmeron et al., 2011; Kruger et al., 2013). Arsenic metabolism and its genetic determinants have been investigated in many Gram-positive and Gram-negative bacteria (Kruger et al., 2013). Bacteria acquired several different arsenic-metabolic strategies including arsenite oxidation (*aio* system) in arsenite oxidizers which oxidize As(III) to As(V) (Oremland and Stolz, 2003; Heinrich-Salmeron et al., 2011), anaerobic arsenate respiration (*arr* system) in arsenate-reducing microbes which respire and reduce As(V) to As(III) (Oremland and Stolz, 2003; Páez-Espino et al., 2009), arsenate system (*ars* system) in arsenate-resistant microbes which reduce cytoplasmic toxic As(V) to As(III) (Oremland and Stolz, 2003; Kruger et al., 2013), and arsenic methylation (*arsM* system) in arsenic methylating bacteria which convert inorganic arsenic into methylated arsenic (Qin et al., 2006; Kruger et al., 2013; Zhang et al., 2015). Arsenic-metabolic genes are assembled with multiple arsenic operons in some bacteria. For example, both *arr* and *ars* operons are found in *Shewanella* sp. (Saltikov and Newman, 2003); *Thiomonas* sp. possess two operons (*aio* and *ars* system) (Arsène-Ploetze et al., 2010); *Herminiimonas arsenicoxydans* has *aio* and *ars* (Muller et al., 2007); *Cyanidioschyzon* sp. 5508 has *aio, arr*, and *ars* operons (including *arsM*) (Qin et al., 2009). Arsenic-resistant microorganisms possibly benefited from multiple arsenic-resistance operons, e.g., they can utilize different detoxification strategies under the complex environments.

With extraordinary metabolic versatility, *R. palustris* has been widely studied for wastewater treatment, bioremediation, hydrogen production and electricity generation (Zhao et al., 2011; Fu et al., 2014; Liu et al., 2015). *R. palustris* is possibly exposed to a high concentration of arsenic under the above field application conditions. Despite considerable research on the arsenic-metabolic mechanisms in chemotrophic bacteria, only few studies have been conducted in anoxygenic phototrophic bacteria. *R. palustris* CGA009 has three arsenic-resistance operons (*ars1, ars2*, and *ars3*) in the chromosome (Qin et al., 2006). Deciphering the arsenic resistance mechanism(s) in anoxygenic phototrophic bacteria may facilitate engineering stronger arsenic resistant strains with desirable features in industrial applications. The objectives of the present study were as follows: (i) to examine the arsenic resistance capacity; (ii) to exam the differential regulation of the three arsenic operons; (iii) to analyze the arsenic speciation in anoxygenic phototrophic bacteria by using *R. palustris* CGA009 as a model organism; (iv) to propose a working model for arsenic resistance in *R. palustris* CGA009.

## Materials and methods

### Bacterial strains, media, and growth conditions

*R. palustris* CGA009 (ATCC BAA-98) was obtained from the American Type Culture Collection (ATCC, USA). Bacteria were anaerobically grown in modified Ormerod medium at 30°C with continuous illumination (Zhao et al., 2011). Briefly, ammonium sulfate and DL-malic acid were substituted by 10 mM of ammonium chloride, 30 mM of sodium acetate, 10 mM of sodium succinate, 10 mM of sodium pyruvate, 10 mM of sodium malate, 10 mM of sodium bicarbonate and 0.1% yeast extract (Oxoid, UK), pH 6.8.

### Arsenic resistance determination

Cells in the log-phase were dispensed into 20-ml screw cap test tube. Various concentrations of Na_3_AsO_4_·12H_2_O and NaAsO_2_ (Merck, Germany) were added with final concentrations ranging from 0.5 to 6.0 mM and ranging from 0.5 to 2.5 mM, respectively. Cell growth was estimated by measuring the optical density at 660 nm (*OD*_*660*_) after incubation anaerobically at 30°C and with 2500 lux light for 4 days (Carius et al., 2013). The starting *OD*_*660*_ was 0.09. The growth index was defined as the median effective concentration (*EC*_50_) and was used to assess the arsenic tolerance in *R. palustris*.

### Determination of arsenic speciation

The process of determination of arsenic speciation as described previously (Lin et al., 2014). Freeze-dried samples were extracted with 10 ml of 1% nitric acid (Merck, Germany) in a microwave-accelerated reaction system (CEM Microwave Technology, UK). This system was provided with a stably increasing temperature from 55 to 75°C within 10 min. Then the extracts were heated at 95°C for 30 min. Finally, the extracted solutions were centrifuged and passed through a nylon filter with a size of 0.22 μm. Arsenic speciation of cells was determined using the high-performance liquid chromatography (HPLC) (Agilent 1200, Japan) coupled with inductively-coupled plasma mass spectrometry (ICP-MS) (Agilent 7500cx, USA) as described previously (Lin et al., 2014). The mobile phases consisted of 6.67 mM of NH_4_H_2_PO_4_ (Merck, Germany) and 6.67 mM of NH_4_NO_3_ (Merck, Germany) at pH 6.2. Arsenic speciation in the samples were identified by comparing their retention times to the standards including arsenite, arsenate, dimethylarsinic acid (DMA) (Chem Service, PA, USA) and monomethylarsonic acid (MMA) (Beijing Chemicals, China).

### Molecular manipulation

Genomic DNA was extracted using the One-4-all Genomic DNA Mini-Preps Kit (Sangon, China). Total RNA was extracted and purified by using an EasyPure™ RNA Kit (TransGen, China). The contaminating DNA was removed by DNase I procedure (Takara, China) if necessary. DNA-free RNA samples were confirmed by PCR amplification of the house-keeping gene *gyrB* (encoding DNA gyrase subunit B) with the forward primer *gyrBF* (AACTGAACGGCATTATGG) and the reverse primer *gyrBR* (GGGATGTTGTTGGTGAAG). cDNAs were synthesized using an PrimeScript™ II 1st strand cDNA synthesis kit (Takara, China) following the protocol. Prior to quantitative PCR, cDNAs were diluted 10-fold as template in nuclease-free water.

Functional genes *arsB, arsC2* and *arsM* were chosen as representative genes for *ars1, ars2*, and *ars3* operons in this study, respectively. *arsB* was amplified with *arsBF* (GCTGAT CGTTTCCAACCT) and *arsBR* (ACCATCACCGAGGCATAA); *arsC2* was amplified with *arsCF* (CGGGCACTTCAGATACTC) and *arsCR* (CGTCGTCATCTATCACAAC); *arsM* was amplified with *arsMF* (CCGACAGGTTGATGACGCAGT) and *arsMR* (CGTCGTCAT CTATCACAAC). *gyrB* gene was used to normalize the expression for *arsB, arsC2*, and *arsM* as described by Saltikov et al. (2005). General PCR experiments were conducted in a T-100 Thermal Cycler (Bio-Rad). Quantitative real-time PCR was carried out in the Applied Biosystems 7300 *Real*-*Time PCR* System (Life Technologies). The cycle thresholds (*C*_*T*_) were determined for samples and genomic DNA standards. Diluted CGA009 genomic DNA was used as standard for quantification. For each transcript, the *C*_*T*_ value was converted to a genomic DNA equivalent in copies by comparing the *C*_*T*_ of an unknown sample to standard curves (prepared by using CGA009 genomic DNA).

## Results and discussion

**Figure 1:**
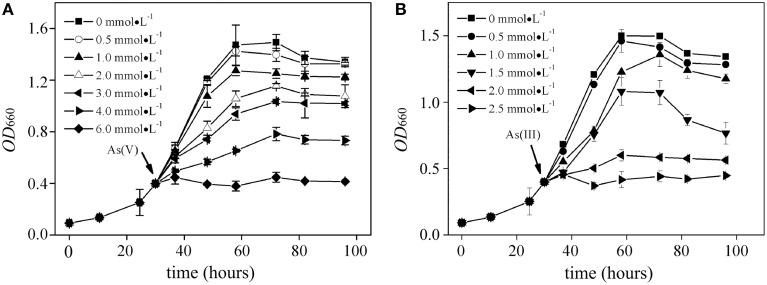
**Growth curves of ***R. palustris*** CGA009 in the presence of different concentrations of arsenate (A) and arsenite (B), respectively**. Strain CGA009 was anaerobically grown at 30°C with continuous illumination. Error bars indicate the standard deviation from three independent experiments.

**Figure 2:**
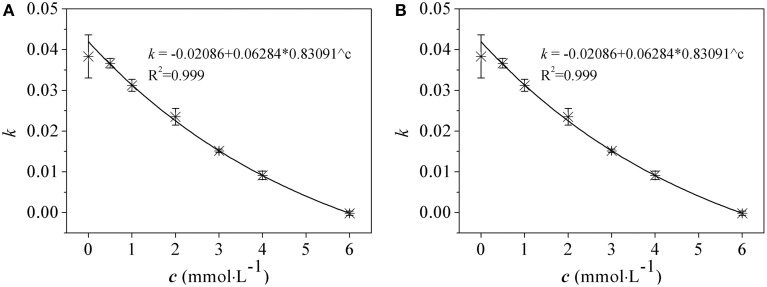
**The relationship between the growth rate and arsenate (A) and arsenite (B) concentrations in ***R. palustris*** CGA009**. Growth rate (*k*) was calculated from the increased OD_*660*_ values in log phase cultures grown in different concentrations of arsenic (*c*). The equations were obtained by nonlinear fitting. *EC*_50_ was obtained from *c* value which corresponded to a half of maximum of *k* value by equations.

Effects of As(V) (0.5~6.0 mM) and As(III) (0.5~2.5 mM) on the bacterial growth of *R. palustris* were tested in modified Ormerod medium and under anaerobic–light conditions (Figure 1). Negligible difference was observed between the culture supplemented with 0.5 mM of As(V) and the control (no AS(V)), indicating that a low concentration of As(V) was not toxic to *R. palustris*. The cells could retain at least 60, 70, and 80% of the control growth when 3.0 mM, 2.0 mM and 1.0 mM of As(V) were added to the culture respectively, indicating a good resistance to As(V). However, higher concentrations of As(V) (i.e., 4.0 mM to 6.0 mM) severely inhibited the bacterial growth with the most significant inhibition (nearly 100%) at the concentration of 6.0 mM. *R. palustris* was more sensitive to environmental As(III) than that in As(V) as shown in Figure 1B. For example, 2.0 mM and 2.5 mM of As(III) inhibited up to 90 and 100% of the bacterial growth, respectively. However, cells retained at least 70 and 60% of the control growth when 1.0 and 1.5 mM of the As(III) were added, respectively. Furthermore, we studied the relationship between the growth rate and the arsenic concentrations (Figure 2). Median effective concentration (*EC*_50_) values for As(V) and As(III) were estimated to be 2.44 and 1.55 mM, respectively.

LV et al. investigated the arsenic resistance in three purple non-sulfur bacteria and reported the *EC*_50_ for As(V) and As(III) *Rhodopseudomona palustris* CQV97 (2.4 and 0.9 mM, respectively), *Rhodobacter azotoformans* 134K20 (1.6 and 0.2 mM, respectively) and *Rhodobacter capsulatus* XJ-1 (1.9 and 0.3 mM, respectively) (Lv et al., 2012a,b). The *EC*_50_-value for arsenic in strain CQV97 was higher or comparable to *E. coli, P. aeruginosa, Rhodococcus equi*, and *Methylosinus trichosporium*. However, compared to those bacteria with a “high” arsenic resistance capability (>100 mM), there is still considerable room for further improvement.

**Figure 3:**
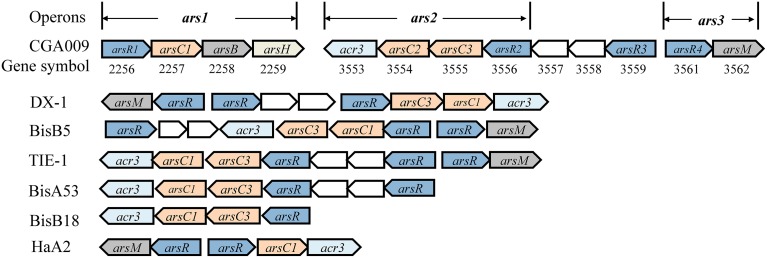
**Schematic comparison of arsenic gene clusters from seven ***R. palustris*** strains**. Genes were shown as different color and arrows indicated the direction of transcription. *arsR* was arsenite-responsive transcriptional regulator gene. *arsC/arsC*' was As (V) reductases gene, *arsC* was As (V) reductases genes, *arsC1 and arsC2* were glutathione (GSH)/glutaredoxin (Grx)-coupled reductases genes, *arsC3* was thioredoxin (Trx)/thioredoxin reductase (TrxR)-dependent reductases gene; *arsB* was arsenite efflux pump gene, *acr3* was arsenite permease (ACR3) gene, they came from unrelated As(III) transporter families; *arsH* was NADPH-dependent flavin mononucleotide reductase gene; *arsM* was As(III)-methyltransferase gene. Other genes found in or adjacent to the *ars* clusters were shown as empty boxes.

The genome-mining analysis showed that *arsRBC* operon existed in seven *R. palustris* strains (CGA009, HaA2, TIE-1, DX-1, BisB5, BisA53, and BisB18) (Figure 3), while additional *arsRM* operon was only found in strains CGA009, HaA2, TIE-1, DX-1, and BisB5 (Figure 3) (Lv et al., 2012a). The first operon resembles *ars* operon (named *ars1* operon, *arsRCBH* type), consisting of arsenite-responsive transcriptional regulator gene (*arsR1*), arsenate reductase gene (*arsC1*), arsenite efflux pump gene (*arsB*) and NADPH-dependent flavin mononucleotide reductase gene (*arsH*). The second operon [named *ars2* operon, *arsRRCC* (*acr3*) type] contains two *arsR* genes (*arsR2* and *arsR3*), two As(V) reductases genes (*arsC2* and *arsC3*), one arsenite permease gene (*acr3*), and two genes encoding the hypothetical proteins. Both *ars1* and *ars2* operons encode these proteins that perform As(V) reduction and As(III) extrusion mechanisms. The third operon (named *ars3* operon, *arsRM* type) consists of arsenite-responsive transcriptional regulator gene (*arsR4*) and As(III)-methyltransferase gene (*arsM*). *ars3* operon encode methylation protein that methylate As(III) to a number of methylated intermediates such as onomethylarsenite [MMA(III)] and dimethylarsenite [DMA(III)]. It should be note that arsenic resistance in microorganisms did not show a direct correlation with arsenic operon number(s) (Kruger et al., 2013). For example, at least five arsenic operons were found in *Herminiimonas arsenicoxydans* while its tolerance to arsenic was much lower than that in *Corynebacterium glutamicum* which has only two arsenic operons. Despite of their widespread distribution in various bacteria, the arsenic multi-operons were understudied. Due to arsenic multi-operons co-existing in one microorganism, it is difficult to define the arsenic resistance mechanisms. Therefore, it is necessary to further investigate how these arsenic multi-operons were differentially regulated and their contributions to arsenic speciation.

**Figure 4:**
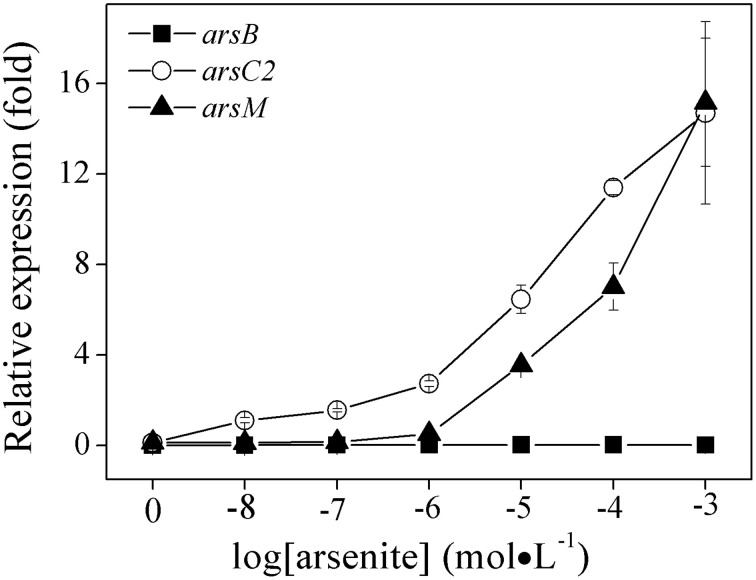
**Effects of As (III) concentrations on the expressions of ***ars1***, ***ars2*** and ***ars3*** operons in ***R. palustris*** CGA009**. The expressions of *arsB*(■), *arsC2*(○), and *arsM*(▴) represent the expression of *ars1, ars2*, and *ars3* operons, respectively. The expression of genes was calculated by determing the content ratio of functional genes to house-keeping gene (*gyrB*). Cultures were harvested in the middle-log phase (50 h). Error bars indicate the standard deviation from three independent experiments.

In this study, we preliminary investigated their respective gene expression in the three *ars* operons by using quantitative real-time PCR in order to understand which one was actively involved in arsenic resistance in *R. palustris* CGA009. It should be noted that studying *ars* operon regulation by As(V) is difficult because it can be quickly reduced to As(III) by As(V) reductase *in vivo*. Therefore, we first examined the effect of As(III) (rather than As(V)) on the expression of *ars*1, *ars*2 and *ars*3 operons. To do that, we selected the functional genes *arsB, arsC2*, and *arsM* as marker genes for *ars*1, *ars*2, and *ars*3 operons, respectively (Figure 4). Gene expression of *arsB, arsC2*, and *arsM* were undetectable when As(III) was absent, indicating the three operons were not expressed without arsenic induction. *arsB* was not transcribed when As(III) was added to the culture at concentrations ranging from 0 to 1.0 mM. Remarkably, the expression of *ars2* operon (*arsC2*) was readably detected when cells were even exposed to a low level of As(III) (0.01 μM). Compared to the control, its transcript level was increased 9.5, 23.8, and 126.8–fold when 0.01, 0.1, and 1.0 mM of As(III) were added respectively, demonstrating that *ars2* expression was up-regulated by As(III). However, *arsM* was not transcribed when the cells were exposed to 1.0 μM of As(III), indicating that gene product of *arsM* was not critical for detoxifying As(III) under low environmental As(III) conditions (<1.0 μM). However, its expression level was 3.86-fold higher than the control when 1.0 μM concentration of As(III) was present in the medium (Figure 4). Furthermore, *arsM* transcript level increased with the increasing environmental As(III) concentration (Figure 4). It should be noted that the expression levels of *arsM* and *arsC2* were almost equal when As(III) concentration reached a 1.0 mM, showing that *R. palustris* CGA009 probably required expression of *ars*2 and *ars*3 operons to detoxify the high concentrations of As(III).

**Figure 5:**
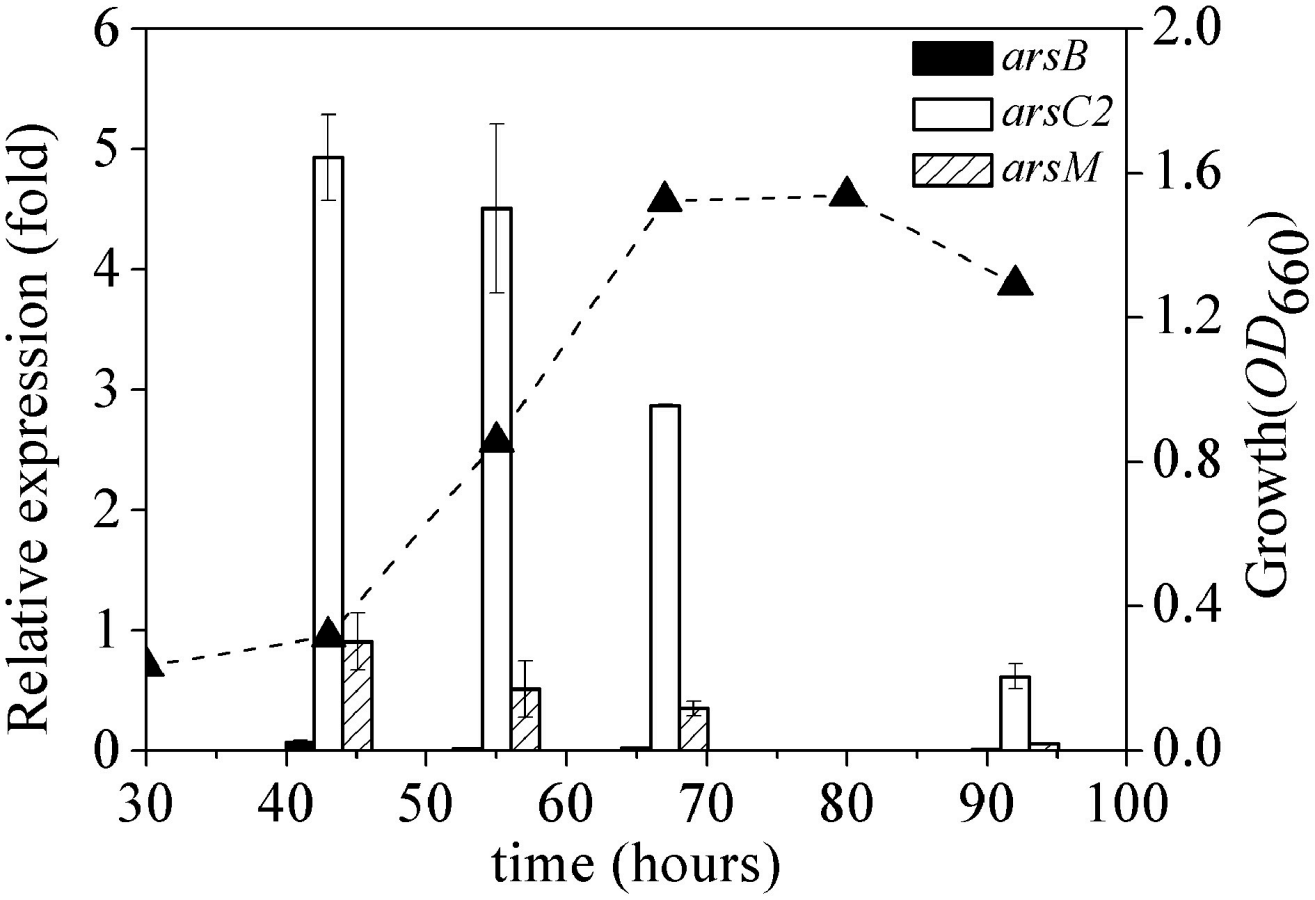
**Dynamics of expression of ***ars1***, ***ars2*** and ***ars3*** operons**. The expression of *arsB* (black bars), *arsC2* (white bars) and *arsM* (bias bars) was normalized to the expression of *gyrB*. The growth curve(▴) of *R. palustris* CGA009 grown on 0.5 mM arsenate is shown as dash line. Error bars indicate the standard deviation from three independent experiments.

The expression dynamics of *arsB, arsC2* and *arsM* (*ars*1, *ars*2, and *ars*3 operons) were investigated in *R. palustris* CGA009 at different phases of growth (Figure 5). *ars*C2 expression was highly induced by arsenate at the log-phase (between 43 and 60 h); the expression level decreased when cells entered the stationary phase (67–80 h). A similar expression pattern was found in *ars*M; however, a relatively low level of expression of *ars*M was recorded during the whole growth phase, compared to that in *arsC2*. *ars1* transcript level was much lower than those in *ars2* and *ars3* at different phases of growth.

Unfortunately, only few studies have been conducted to exam the differential regulation of arsenic multi-operons by arsenic. In bacteria and archaea, *ars* operons are often controlled by ArsR (Figure 3). When As(III) is absent, ArsR binds to the operator/promoter region of the operon and thus repressed the downstream genes' transcription. When As(III) is available, ArsR binds to it and goes through conformational changes, resulting in dissociation from the operator/promoter region. In our transcript analysis experiments, we could not detect a significant *ars*1 expression (Figure 4). However, this observation was not new. For example, in *C. glutamicum*, only *arsC1*' was constitutively transcribed though there were two arsenic resistance operons (*ars1* and *ars2*) (Villadangos et al., 2011). Furthermore, the expression of *ars*3 operon in *R. palustris* CGA009 was induced with the presence of 1.0 μM of As(III), indicating it may only contribute to the arsenic detoxification under the higher concentrations of As(III) (see next). However, the expression pattern was not similar to those previously observed in *Synechocystis* sp. PCC 6803 and *P. alcaligenes* NBRC14159 (López-Maury et al., 2003; Zhang et al., 2015). *Synechocystis* sp. PCC 6803 has at least two operons (*ars* and *arsM*) on its chromosome. *ars* operon was regulated by ArsR though *arsR* was located far away from *ars*. DNase I footprinting experiments indicated that ArsR binds to two 17-bp direct repeats (ATCAA(N)6TTGAT) in the promoter-operator region. However, the upstream sequence of *arsM* does not have the 17-bp repeats (López-Maury et al., 2003). Authors proposed that ArsM is constitutively expressed whilst how ArsM expression is regulated has yet to be investigated. In *P. alcaligenes* NBRC14159, *PaarsM* was expressed in the absence of As(III) and the expression was further enhanced by As(III) exposure (Zhang et al., 2015). Our results revealed that the expression of the different arsenic operons were affected by growth cycle: the maximal expressions of both *ars*2 and *ars*3 operons in CGA009 appeared at early log-phase (43 h) and maintained at high level during the growth of middle log-phase (55 h). It is different from expression patterns reported in the earlier studies in *Shewanella* sp. ANA-3 (Saltikov et al., 2005). The highest *arr* expression appeared at the log-phase while peak expression level of *ars* was observed in the stationary phase, respectively. That transcription of the *ars* operon in *Shewanella* sp. ANA-3 could involve other factors, such as those related to quorum sensing and energy production. In fact, a quorum-sensing-based response was shown to be a second regulatory circuit for *aio* transcription in *A. tumefaciens* (Kashyap et al., 2006).

**Figure 6:**
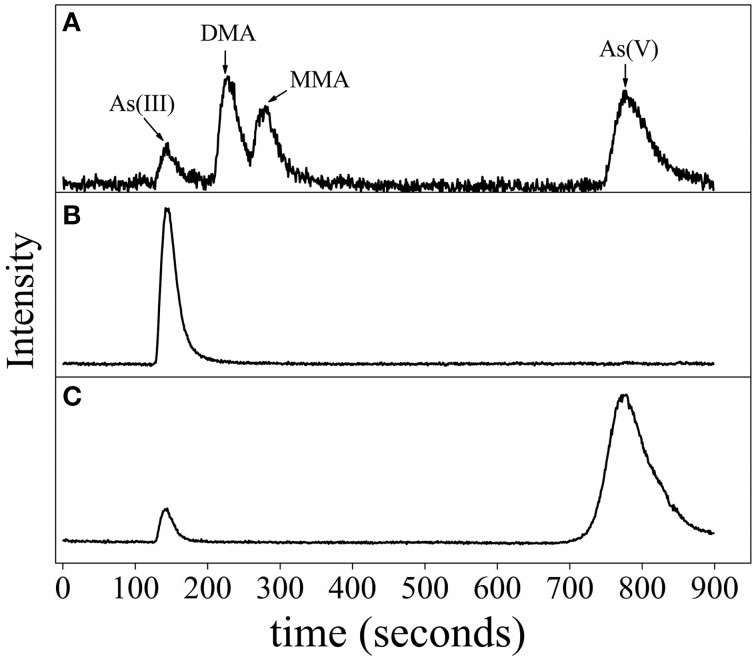
**Arsenic speciation of ***R. palustris*** CGA009 determined by anion exchange HPLC-ICP-MS**. **(A)** Chromatogram of the standards, including As (III), dimethylarsine (DMA), monomethylarsine (MMA) and As (V). **(B)** Speciation of arsenic of *R. palustris* CGA009 grown on 1.0 mM arsenite in the middle-log phase. **(C)** Speciation of arsenic of *R. palustris* CGA009 grown on 0.5 mM arsenate in the middle-log phase.

HPLC-ICP-MS analysis in middle log-phase demonstrated that As(V) was reduced to As(III), indicating that strain CGA009 detoxified arsenate by reducing As(V) to As(III) by ArsC (Figure 6). However, we failed to detect dimethylarsine (DMA), monomethylarsine (MMA) even in the growth stage where *arsM* transcript approached to the highest level (Figure 6). DMA and MMA, the intermediates produced in As(III) methylation process did not accumulate in the cells and were immediately converted into volatile trimethylarsine (TMA). Thereafter, TMA were rapidly expelled to extracellular space (Qin et al., 2006). In the same study, Qin *et al*. heterologously expressed the *arsM* gene from *R. palustris* CGA009 in an arsenic-sensitive strain of *E. coli*. Their results showed that ArsM catalyzed the formation of a number of methylated intermediates from As(III), with TMA as the end product and increased the arsenic resistance, indicating that it was very possible for *arsM* to be functional *in vivo* (Qin et al., 2006).

**Figure 7:**
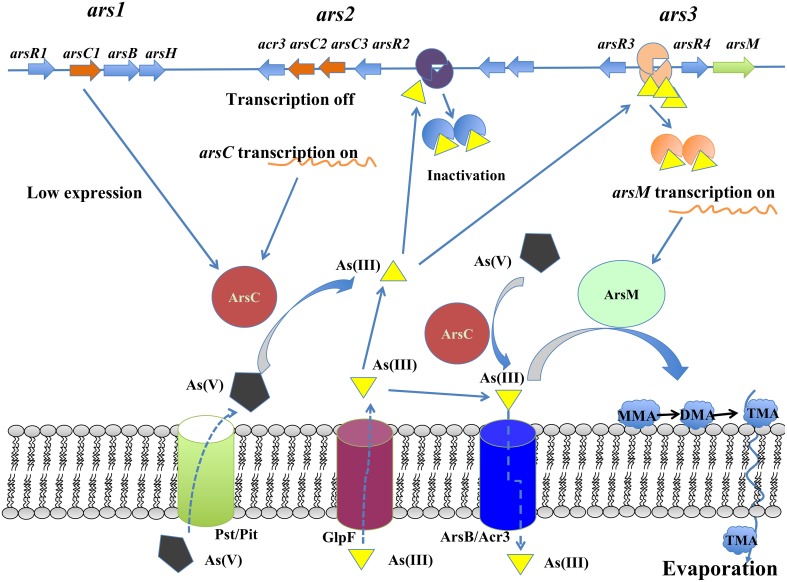
**Proposed arsenic metabolic model for ***R. palustris*** CGA009**.

A working model for the arsenic detoxification by the arsenic multi-operons was proposed in *R. palustris* (Figure 7). In this model, the As(V) enters into cells through inorganic phosphate (Pit) or phosphate specific transport (Pst) systems (Kruger et al., 2013); once As(V) arrives inside the cells, it is reduced to As(III) by ArsC produced from *ars1* and/or *ars2* operon (possible at a low expression) (Figure 3). As(III) formed in the reaction then inactivates ArsR, initiating the transcription of the *arsRRCC* (*acr3*) operon (*ars2*). Due to the two copies of *arsC* in *ars2*, it allows to reduce arsenate more promptly, leading to As(III) accumulation in the cells. If the accumulated As(III) could not be expelled out of the cells by arsenite permease (Acr3), the increasing As(III) triggers the *ars3* transcription by releasing ArsR which originally binds *arsM* promoter/operator. Arsenic resistance genetic units *ars2* and *ars3* in *R. palustris* contribute to detoxifying arsenic at a high dose because their transcription level is comparable when cells are treated with 1.0 mM arsenite. With cooperation of *ars2* and *ars3, R. palustris* detoxifying As(III) by extruding it out of the cell by As(III) transporter (Acr3) or transforming As(III) to volatile methylated As(III) (TMA) by ArsM. It is reasonable to assume that *ars2* is more important when cells are exposed to lower levels of arsenic due to its activity expression. However, a relatively complex arsenate detoxification system described here suggests that *R. palustris* growing in the natural environment must be equipped to deal with rapidly changing and perhaps relatively high levels of arsenic.

## Conclusion

This study provided a novel insight into arsenic resistance mechanisms in *R. palustris* CGA009, a member of anoxygenic phototrophic bacteria. *R. palustris* possessed good arsenic resistance which possibly linked to the arsenic multi-operons in the chromosome. Our results showed *ars2* and *ars3* operons were upregulated by increasing As(III) concentrations while *ars*3 operon was only expressed when exposed to the arsenic concentrations more than 1.0 μM. However, the expression of *ars1* operon was very low, indicating that *ars1* may not be actively used. Collectively, our preliminary data showed that arsenic was possibly transformed by the combination mechanisms of the cytoplasmic As(V) reduction, As(III) extrusion and arsenic methylation when exposed to arsenic at a high concentration.

### Conflict of interest statement

The authors declare that the research was conducted in the absence of any commercial or financial relationships that could be construed as a potential conflict of interest.
